# A Rare Deep-Rooting D0 African Y-Chromosomal Haplogroup and Its Implications for the Expansion of Modern Humans Out of Africa

**DOI:** 10.1534/genetics.119.302368

**Published:** 2019-06-13

**Authors:** Marc Haber, Abigail L. Jones, Bruce A. Connell, Asan, Elena Arciero, Huanming Yang, Mark G. Thomas, Yali Xue, Chris Tyler-Smith

**Affiliations:** * The Wellcome Sanger Institute, Hinxton, Cambridgeshire CB10 1SA, UK; † Liverpool Women’s Hospital, Liverpool L8 7SS, UK; ‡ Glendon College, York University, Toronto, Ontario M4N 3N6, Canada; § BGI-Shenzhen, Shenzhen 518083, China; ** James D. Watson Institute of Genome Science, 310008 Hangzhou, China; †† Research Department of Genetics, Evolution and Environment, University College London, WC1E 6BT, UK, and University College London (UCL) Genetics Institute, University College London, WC1E 6BT, UK

**Keywords:** Human Y chromosome, YAP+ Y chromosomes, phylogeography, out-of-Africa migration

## Abstract

Present-day humans outside Africa descend mainly from a single expansion out ∼50,000–70,000 years ago, but many details of this expansion remain unclear, including the history of the male-specific Y chromosome at this time. Here, we reinvestigate a rare deep-rooting African Y-chromosomal lineage by sequencing the whole genomes of three Nigerian men described in 2003 as carrying haplogroup DE* Y chromosomes, and analyzing them in the context of a calibrated worldwide Y-chromosomal phylogeny. We confirm that these three chromosomes do represent a deep-rooting DE lineage, branching close to the DE bifurcation, but place them on the D branch as an outgroup to all other known D chromosomes, and designate the new lineage D0. We consider three models for the expansion of Y lineages out of Africa ∼50,000–100,000 years ago, incorporating migration back to Africa where necessary to explain present-day Y-lineage distributions. Considering both the Y-chromosomal phylogenetic structure incorporating the D0 lineage, and published evidence for modern humans outside Africa, the most favored model involves an origin of the DE lineage within Africa with D0 and E remaining there, and migration out of the three lineages (C, D, and FT) that now form the vast majority of non-African Y chromosomes. The exit took place 50,300–81,000 years ago (latest date for FT lineage expansion outside Africa – earliest date for the D/D0 lineage split inside Africa), and most likely 50,300–59,400 years ago (considering Neanderthal admixture). This work resolves a long-running debate about Y-chromosomal out-of-Africa/back-to-Africa migrations, and provides insights into the out-of-Africa expansion more generally.

## Abstract (precis)

Humans expanded out of Africa 50,000-70,000 years ago, but many details of this migration are poorly understood. Here, Haber *et al.* sequence Y chromosomes belonging to a rare African lineage and analyze...

HUMANS outside Africa derive most of their genetic ancestry from a single migration event 50,000–70,000 years ago, according to the current model supported by genetic data from genome-wide (Mallick *et al.* 2016; Pagani *et al.* 2016), mitochondrial DNA (mtDNA) (van Oven and Kayser 2009), and Y-chromosomal (Wei *et al.* 2013; Hallast *et al.* 2015; Karmin *et al.* 2015; Poznik *et al.* 2016) analyses. The migrating population carried only a small subset of African genetic diversity, particularly strikingly for the nonrecombining mtDNA and Y chromosome where robust calibrated high-resolution phylogenies can be constructed, and in each case all non-African lineages descend from a single African lineage, L3 for mtDNA or CT-M168 for the Y chromosome. Yet there has been a long-running debate about the early spread of Y-chromosomal lineages because their current distributions do not fit a simple phylogeographical model. The CT-M168 branch diverged within a short time interval into three lineages (C-M130, DE-M145, and FT-M89), and just a few thousand years later the lineage DE-M145 further split into D-M174 and E-M96 (Poznik *et al.* 2016), illustrated in Supplemental Material, Figure S1. Thus, around the time of the expansion out of Africa, between one (CT-M168) and four (C-M130, D-M174, E-M96, and F-M89) of the known extant non-African lineages were in existence (plus additional African lineages). The complexity arises because three of these four early lineages (C-M130, D-M174, and FT-M89) are exclusively non-African, apart from those entering Africa through recent gene flow; while the fourth lineage (E-M96) is largely African, where it constitutes the major lineage in most African populations. The debate began in the absence of reliable calibration, and these distributions were interpreted as arising in two contrasting ways: (1) an Asian origin of DE-M145 (also known as the YAP+ lineage), implying migration of CT-M168 out of Africa followed by divergence into the four lineages outside Africa and then migration of E-M96 back to Africa (Altheide and Hammer 1997; Hammer *et al.* 1998; Bravi *et al.* 2000), or (2) an African origin of DE-M145, implying divergence of CT-M168 within Africa followed by migration of C-M130, D-M174, and FT-M89 out (Underhill and Roseman 2001; Underhill *et al.* 2001). The first scenario requires two intercontinental lineage migrations, while the second requires three and is thus slightly less parsimonious.

An additional very rare haplogroup, DE*, carrying variants that define DE but none of those that define D or E individually, added to this complexity. First identified in 5 out of 1247 Nigerians within a worldwide study of >8000 men (Weale *et al.* 2003), DE* chromosomes were subsequently reported in a single man among 282 from Guinea-Bissau in West Africa (Rosa *et al.* 2007) and in 2 out of 722 Tibetans within a study of 5783 East Asians (Shi *et al.* 2008). While the phylogeographic significance of these rare lineages was immediately recognized, their interpretation was hindered by the incomplete resolution of the phylogenetic branching pattern and the possibility that they might originate from back-mutations at the small numbers of variants used to define the key D and E haplogroups, or genotyping errors rather than representing deeply divergent lineages, plus the lack of a robust timescale. Large-scale sequencing of Y chromosomes has now provided both the phylogenetic resolution and the timescale needed (Wei *et al.* 2013; Hallast *et al.* 2015; Karmin *et al.* 2015; Poznik *et al.* 2016), so we have therefore reinvestigated the original Nigerian DE* chromosomes using whole-genome sequencing to clarify their phylogenetic position. We then consider the implications for the out-of-Africa/back-to-Africa debate related to Y-chromosomal lineages, and the expansion out of Africa more generally.

## Materials and Methods

### Samples and sequencing

We analyzed five DE* samples described previously (Weale *et al.* 2003), in the context of published worldwide Y-chromosomal sequences including Japanese D and many E Y chromosomes (Mallick *et al.* 2016). We also included four haplogroup D samples from Tibet (Xue *et al.* 2006), which were newly sequenced for this study; the Japanese and Tibetan D chromosomes represent the deepest known split within D, since Andamanese D chromosomes lie on the same branch as the Japanese (Mondal *et al.* 2017).

Sequencing of the Nigerian samples was carried out at the Wellcome Sanger institute on the Illumina HiSeq X Ten platform (paired-end read length 150 bp) to a Y-chromosome mean coverage of ∼16×. Sequences were processed using biobambam version 2.0.79 to remove adapters, mark duplicates, and sort reads. bwa-mem version 0.7.16a was used to map the reads to the *hs37d5* reference genome. We found that two pairs of individuals were likely duplicates (Figure S2) and thus one of each pair was removed, leaving three Nigerian individuals for further analysis. The four individuals from Tibet were sequenced in the same way to a Y-chromosome mean coverage of ∼18×.

For comparative data from other haplogroups, we obtained Y-chromosome bam files for 173 males representing worldwide populations from the Simons Genome Diversity Project (Mallick *et al.* 2016).

### Data analysis

Y-chromosome genotypes were called jointly from all 180 samples using FreeBayes v1.2.0 (Garrison and Marth 2012) with the arguments “–report-monomorphic” and “–ploidy 1.” Calling was restricted to 10.3 Mb of the Y chromosome previously determined to be accessible to short-read sequencing (Poznik *et al.* 2013). Then sites with depth across all samples <1900 or >11,500 (corresponding to DP/2 or DP*3), or missing in >20% of the samples, were filtered. In individuals, alleles with DP <5 or GQ <30 were excluded, and if multiple alleles were observed at a position, the fraction of reads supporting the called allele was required to be >0.8.

Genome-wide genotypes from the Nigerian samples were called using BCFtools version 1.6 (bcftools mpileup -C50 -q30 -Q30 | bcftools call -c), then merged with data from ∼2500 people genotyped on the Affymetrix Human Origins array (Patterson *et al.* 2012; Lazaridis *et al.* 2016). Principal Component Analysis (PCA) using genome-wide SNPs was performed using EIGENSOFT v7.2.1 (Patterson *et al.* 2006) and plotted using R (R Core Team 2017).

We inferred a maximum likelihood phylogeny of Y chromosomes using RAxML v8.2.10 (Stamatakis 2014) with the arguments “-m ASC_GTRGAMMA” and “–asc-corr=stamatakis,” using only variable sites with QUAL ≥1, and selecting the tree with the best likelihood from 100 runs, then replicating the tree 1000 times for bootstrap values. The tree was plotted using Interactive Tree Of Life (iTOL) v3 (Letunic and Bork 2016) and annotated with haplogroup names assigned using yHaplo (Poznik 2016) from SNPs reported by the International Society of Genetic Genealogy (ISOGG v11.01).

The ages of the internal nodes in the tree were estimated using the ρ statistic (Forster *et al.* 1996), the standard approach for the Y chromosome. We defined the ancestral state of a site by assigning alleles as ancestral when they were monomorphic in the nine samples belonging to the A and B haplogroups in our data set. We then determined the age of a node as follows: Having an ancestral node leading to two clades, we select one sample from each clade and divide the number of derived variants found in the first sample but absent from the second, by the total number of sites having the ancestral state in both samples. We compare all possible pairs under a node and report the average value of divergence times in units of years by applying a point mutation rate of 0.76 × 10^−9^ mutations per site per year (Fu *et al.* 2014). We report 95% confidence intervals of the divergence times based on the 95% highest posterior density when estimating the mutation rate (0.67–0.86 × 10^−9^) (Fu *et al.* 2014). This model assumes that mutations accumulated on the chromosomes in the different lineages at similar rates, and thus expects all individuals in our data set to have comparable branch lengths from the AB root. But we found considerable differences among individuals in the number of their derived mutations from the root. This heterogeneity in the accumulation of mutations has been previously reported (Scozzari *et al.* 2014; Barbieri *et al.* 2016) and appears to be haplogroup-specific (Figure S3), and therefore in our divergence time estimates, we calibrate all lineages to have identical branch length from the root, equal to the average branch length estimated from all individuals in our data set. We first calculated the average number of mutations which accumulated on the branches of all individuals in our data set and found 768.59 derived mutations on average from the root (corresponding to ∼100,000 years). We then derived a calibration coefficient α for each individual by dividing 768.59 by the normalized (in 10,000,000 bp) number of derived mutations an individual has accumulated from the root. And thus for calibrating the branches’ length between any two samples when calculating the split times, we multiply α by the number of derived variants found in the first sample but absent from the second.

### Data availability statement

New sequence data from the Nigerian samples are available through the European Genome-phenome Archive (EGA) under study accession number EGAS00001002674, and for Tibetan samples under study accession number EGAS00001003500.

Three supplemental figures and two supplemental tables accompany this paper:

Figure S1 Y-chromosomal phylogeny as understood before the current study.
Figure S2 PCA of worldwide populations.
Figure S3 Number of mutations from the AB root.
Table S1 SNPs defining the D0 haplogroup.
Table S2 Split-time estimates using the ρ statistic.
Supplemental material available at FigShare: https://doi.org/10.25386/genetics.8267861.

## Results

### Construction of a calibrated Y-chromosomal phylogeny

**Figure 1:**
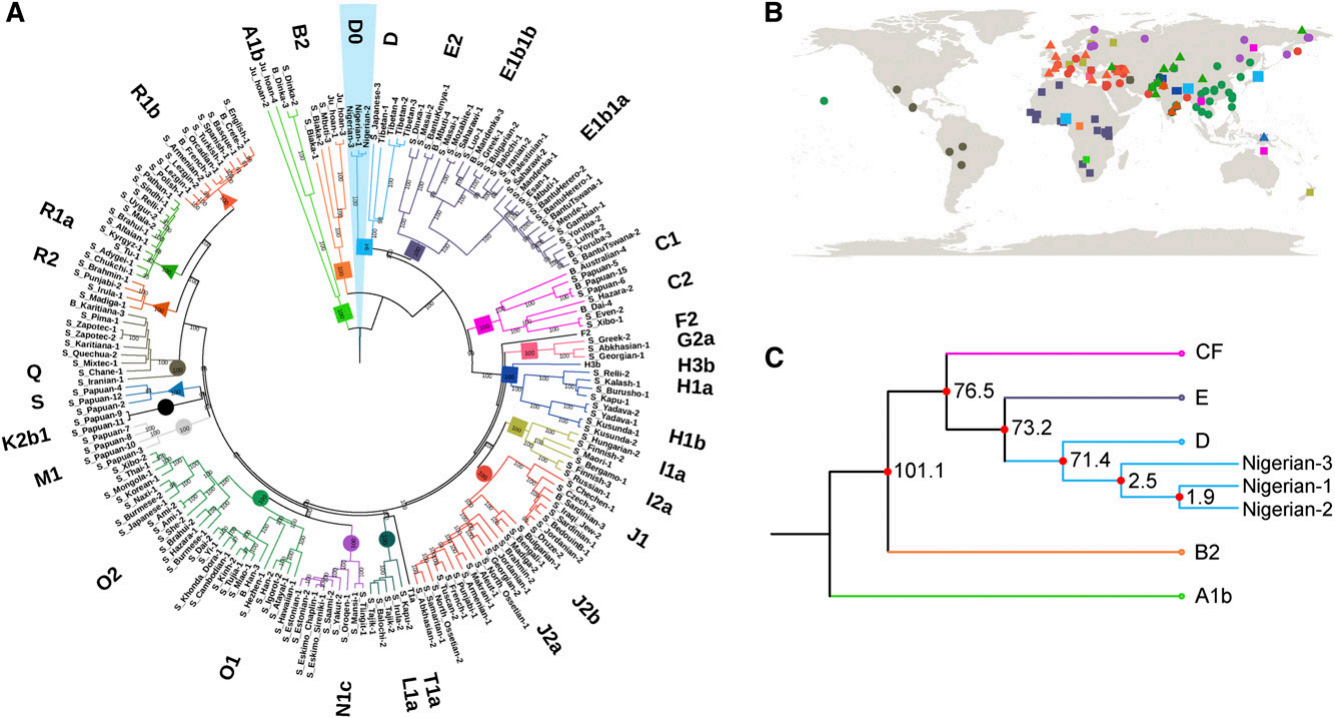
Y Chromosome phylogenetic tree from worldwide samples. (A) A maximum-likelihood tree of 180 Y-chromosome sequences from worldwide populations. Different branch colors and symbols represent different haplogroups assigned based on ISOGG v11.01. The Nigerian chromosomes sequenced in this study are highlighted in blue and assigned to the novel D0 haplogroup. Bootstrap values from 1000 replications are shown on the branches. (B) Map showing location of the studied individuals with colored symbols reflecting the haplogroups assigned in A. The clade consisting of the D0 and D haplogroups is represented by blue squares and is observed in Africa and East Asia. (C) Ages of the nodes leading to haplogroup D0 in the phylogenetic tree (point estimates; branch lengths are not to scale). Haplogroups D0 and D are estimated to have split 71,400 (63,100–81,000) years ago while the D0 individuals in this study coalesced 2500 (2200–2800) years ago.

We constructed a series of phylogenetic trees based on all the Y-chromosomal sequences in our data set, or subsets of them. All showed a consistent structure, in which the Nigerian DE* chromosomes formed a clade branching from the DE lineage close to the divergence of D and E chromosomes (Figure 1A) in comparison with a set of Y chromosomes representing most of the world (Figure 1B). The Nigerian chromosomes had 489 derived SNPs exclusive to their branch in addition to a large deletion spanning ∼118,000 bp (Y:28,457,736–28,576,276). All DE-M154 chromosomes shared 29 SNPs. The Nigerian chromosomes shared seven SNPs with other D chromosomes, one SNP with E chromosomes, one SNP with C1b2a chromosomes, and one SNP with an F2 chromosome (Table S1). The reads overlapping these SNPs were visually investigated using the Integrative Genomics Viewer (IGV) version 2.4.10 and seen to support the calls. We consider sharing of a single SNP as a recurrent mutation in different lineages and interpret the Nigerian chromosomes as lying on the D lineage, diverging from other D chromosomes at 71,400 years ago (Figure 1C), very soon after its divergence from E at 73,200 years ago. We name the lineage formed by the Nigerian samples D0, to reflect its position on the tree and avoid the need to rename all the other D lineages.

The three D0 chromosomes are distinguishable from one another, and have a coalescence time of ∼2500 years (Figure 1C), consistent with their collection from different villages, languages, ethnic backgrounds, and paternal birthplaces (Weale *et al.* 2003). The autosomal genomes of these individuals confirm their genetic ancestry as West Africans (Figure S2).

### Models for the expansion of Y-chromosomal lineages out of Africa

The updated phylogeny including the D0 lineage adds two key pieces of information to the debate about the phylogeography of the Y lineages ∼50,000–100,000 years ago and the mode of expansion out of Africa. First, it increases the number of relevant lineages at this early time period from four to five, and second, it provides a reliable timescale for the branching times of these lineages, and thus for the lineages in existence at any particular time point.

**Figure 2:**
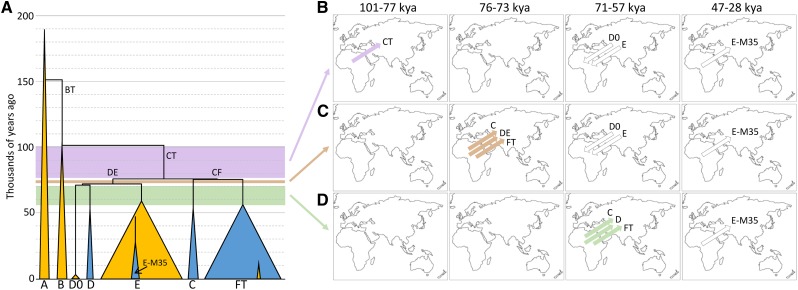
Models for the early movements of Y-chromosomal haplogroups out of Africa and back. (A) Simplified Y-chromosomal phylogeny showing the key lineages, including D0. Lineages currently located in Africa are colored yellow; those currently outside Africa are blue. Triangle widths are not meaningful, except that they show that E and FT are the predominant lineages inside and outside Africa, respectively. The small orange triangle in FT represents the R1b-V88 back-to-Africa migration that took place after the time period considered here. (B–D) Models for lineage movements that could lead to the present-day African or non-African distributions of lineages, using point estimates of dates derived from the phylogeny (see Table S2). The three models represent migrations out of Africa at different time intervals, indicated by the purple, brown, and green shading in A. Arrows in B–D indicate intercontinental movements and their direction, but do not represent particular locations or routes. The first colored arrow(s) represent the lineage(s) that migrated out, during the time period shown at the top of the maps. Additional uncolored arrows represent subsequent migrations and their time intervals needed to produce the present-day distributions.

In the phylogeny (Figure 1A), the DE lineage now contains three, rather than two, early sublineages: one exclusively African (D0), one mainly African (E), and one exclusively non-African (D). We therefore consider the implications of this revised structure for interpreting the present-day Y phylogeography as the result of male movements at different times between 28,000 and 100,000 years ago (Figure 2). To do this, we need to calibrate the phylogeny, and for this use the ancient-DNA-based mutation rate (Fu *et al.* 2014), which has been widely adopted (*e.g.*, Poznik *et al.* 2016); we consider in the *Discussion* the implications of alternative mutation rates and some of the other simplifying assumptions we make here.

We consider three scenarios based on our split-time point estimates of the Y-chromosomal lineages (Table S2). First, between 101,000 years ago (divergence of the B and CT lineages) and 77,000 years ago (divergence of the DE and CF lineages) only one lineage with present-day non-African descendants is present in the phylogeny (CT; Figure 2A), so present-day Y-lineage distributions could be explained by migration of the single lineage CT out of Africa, followed by back-migration of the D0 and E lineages between 71,000 years ago (origin of D0) and 59,000 years ago (divergence of E within Africa) (Figure 2B). This and all other scenarios require migration out of E-M35 after 47,000 years ago (its origin) and before 28,500 years ago (its divergence) to explain its presence outside Africa (Figure 2, B–D). Second, between 76,000 years ago (divergence between C and FT) and 73,000 years ago (divergence between D and E), three relevant lineages are present (the C, DE, and FT lineages, Figure 2A), so migration out of these three followed by back-migration of D0 and E as above (Figure 2C) would explain the distributions. Third, between 71,000 years ago (split of D and of D0) and 57,000 years ago (divergence within FT), five relevant lineages are present, and migration out of three of these (C, D, and FT) would explain the present-day distributions without requiring back-migration (Figure 2D). For simplicity, we do not include the short intervals between these three scenarios of 500 years and 1800 years (Figure 2A and Table S2).

## Discussion

The new D0 data presented in this work are based on just three Y chromosomes, but have far-ranging implications for the structure of the Y-chromosomal phylogeny and hence male movements and migration out of Africa more generally. Our phylogenetic results are consistent with three scenarios (Figure 2, B–D), and we now consider some of the complexities associated with these, and how they fit with nongenetic data.

Complexities arise because although the phylogenetic structure, including the branching order, is very robust (Wei *et al.* 2013; Hallast *et al.* 2015; Karmin *et al.* 2015; Poznik *et al.* 2016), its calibration depends entirely on the mutation rate used. The mutation rate chosen above, based on the number of mutations “missing” in a 45,000-year-old Siberian Y chromosome (Fu *et al.* 2014), has been widely adopted (Poznik *et al.* 2016; Balanovsky 2017), but a large-scale study of Icelandic pedigrees encompassing the last few centuries suggested a rate ∼14% faster (Helgason *et al.* 2015). This faster mutation rate would translate directly into 14% more recent time estimates so that, for example, the Y-chromosome movements out of Africa in the three scenarios presented above would be 87,000–66,000, 65,000–63,000, and 61,000–49,000 years ago, respectively. These differences between mutation rates inferred in different ways should be seen within the context of a wider debate about human mutation rates, previously based largely on autosomal data (Scally and Durbin 2012). Each mutation rate is also accompanied by its own uncertainty, leading to the 95% confidence intervals in Table S2, which include the mutation rate uncertainty. We also assume that the mutation rate is constant over time and does not differ between lineages. The first assumption is very reasonable for the time period of most interest here, 50,000–60,000 years, when the mutation rate averaged over 45,000 years (Fu *et al.* 2014) is used. A flexible mutation rate that assumed a real increase in recent times would have little influence on these estimates since the Fu *et al.* rate already includes the last few centuries. Differences in mutation rate between lineages need further investigation, but would not be sufficient to affect the scenarios presented in Figure 2. For these reasons, we believe that the Fu *et al.* rate, averaged over 45,000 years, is the appropriate one to use for the times of interest here.

These genetic times can be compared with dates from nongenetic sources for modern humans outside Africa. The 45,000-year-old Siberian fossil (Fu *et al.* 2014) was reliably dated using carbon-14, while a ∼43,000-year-old fragment of human maxilla from the Kent’s Cavern site in the UK was dated using Bayesian modeling of stratigraphic, chronological, and archaeological data (Higham *et al.* 2011). Archaeological deposits at Boodie Cave in Australia were dated to ∼50,000 years ago using optically stimulated luminescence (Veth *et al.* 2017). Thus, there is strong support for the widespread presence of modern humans outside Africa 45,000–50,000 years ago. Earlier dates have also been reported, for example the Madjedbebe rock shelter in northern Australia dated by optically stimulated luminescence to at least 65,000 years ago (Clarkson *et al.* 2017), a modern human cranium from Tam Pa Ling, Laos was dated by Uranium-Thorium to ∼63,000 years ago (Demeter *et al.* 2012), and 80 teeth from Fuyan Cave in southern China dated using the same method to 80,000–120,000 years ago (Liu *et al.* 2015), raising the possibility of a substantially earlier exit (Bae *et al.* 2017). Such early archaeological dates also, however, raise the question of whether or not the humans associated with them contributed genetically to present-day populations (Mallick *et al.* 2016; Pagani *et al.* 2016). Archaeological data alone therefore do not provide an unequivocal date for the migration of the ancestors of present-day humans out of Africa.

**Figure 3:**
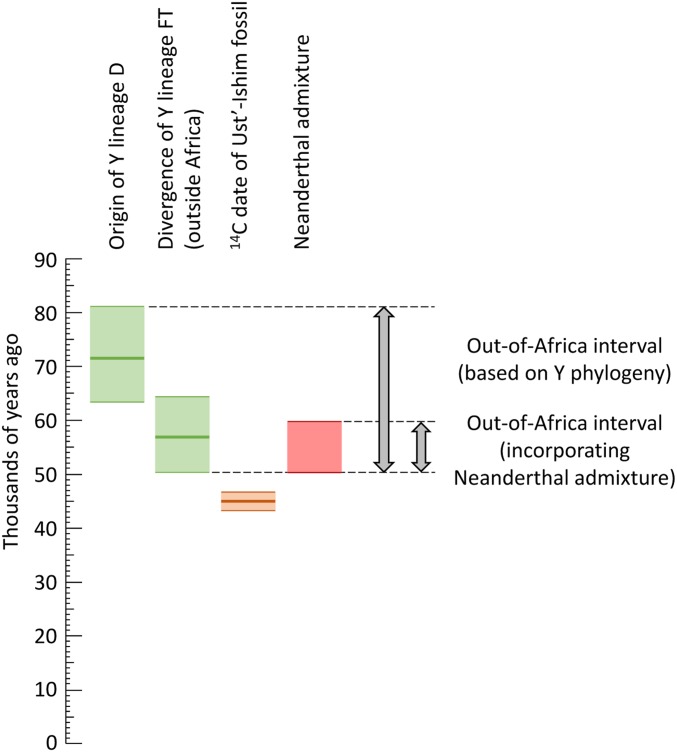
Estimation of the time of the out-of-Africa migration incorporating information from Y-chromosomal lineages (green, this work), archaeological dates (brown, Fu *et al.* 2014), and ancient DNA (red, Fu *et al.* 2014).

All non-Africans carry ∼2% Neanderthal DNA in their genomes (Green *et al.* 2010), and Neanderthal fossils have only been reported outside Africa. The geographical distribution of Neanderthals thus suggests that mixing probably occurred outside Africa, and the ubiquitous presence of Neanderthal DNA in present-day non-Africans is most easily explained if the mixing took place once, soon after the migration out. This mixing has been dated with some precision using the length of the introgressed segments in the 45,000-year-old (43,210–46,880 years) Siberian male (Ust’-Ishim) to 232–430 generations before he lived, *i.e.*, 49,900–59,400 years ago assuming a generation time of 29 years (Fu *et al.* 2014). If this date represented the time of the migration out of Africa, it would exclude the first two scenarios (Figure 2, B and C). Thus, the combination of Y phylogenetic structure and dating of the out-of-Africa migration based on the 45,000-year-old Siberian fossil (Fu *et al.* 2014) favors the third scenario (Figure 2D) involving the migration out of C, D, and FT between 50,300 years ago (lower bound of the FT diversification, Table S2) and 59,400 years ago (upper bound of the introgression; see Figure 3), which is in accordance with suggested models incorporating an African origin of the DE lineages (Underhill and Roseman 2001; Underhill *et al.* 2001). According to this interpretation, the reported Tibetan DE* chromosomes (Shi *et al.* 2008) would most likely represent back-mutations or genotyping errors at the one SNP used to define haplogroup D, but require further investigation.

mtDNA sequences also provide a robust phylogeny which demonstrates that non-African mtDNAs descend from a single African branch with rapid diversification outside Africa into the M and N lineages and many subsequent branches (Ingman *et al.* 2000; Devièse *et al.* 2019). Dating using ancient mtDNA suggests a separation of non-African from African lineages after 62,000–95,000 years ago (Fu *et al.* 2013), while an analysis of present-day mtDNAs suggested divergence outside Africa 57,000–65,000 years ago (Fernandes *et al.* 2012). These estimates are based on <1% of the sequence length used from the Y chromosome but are nonetheless very consistent.

This discussion has thus far assumed that present-day distributions of Y haplogroups are relevant to events 50,000–100,000 years ago and thus that Y phylogeography carries information about the major migration out of Africa. Ancient population structure within Africa that separated C, D, and FT from other Y haplogroups beginning after 76,000 years ago with migration out only 50,000–59,000 years ago would also fit the evidence presented above. Present-day Y-chromosomal structure in Africa has been massively shaped by events in the last 10,000 years, including the Bantu-speaker expansion in central and southern Africa (Poznik *et al.* 2016; Patin *et al.* 2017) and entry of Eurasian lineages into northern and central Africa (Haber *et al.* 2016; D’Atanasio *et al.* 2018), and is thus a poor guide to structure before 10,000 years ago. Despite this, it is striking that western central Africa is the location of the deepest-rooting A00 lineage in Cameroon (Mendez *et al.* 2013), a major location of the A0 lineage in Cameroon, The Gambia, and Ghana (Scozzari *et al.* 2014; Poznik *et al.* 2016) and the D0 lineage in Nigeria and Guinea-Bissau (Weale *et al.* 2003; Rosa *et al.* 2007). This retention of the deepest Y-chromosomal diversity in western central Africa contrasts with the autosomal genetic structure, where the deepest roots have been reported in southern African hunter-gatherers (Gronau *et al.* 2011; Schlebusch *et al.* 2012, 2017; Veeramah *et al.* 2012; Mallick *et al.* 2016; Skoglund *et al.* 2017), perhaps supporting the hypothesis of deep population structure (Henn *et al.* 2018; Scerri *et al.* 2018). Analysis of ancient African DNA from 50,000 to 100,000 years ago would provide considerably more information on Y-haplogroup distributions at this time, but is not currently available. In the meantime, further focus on present-day Y-chromosomal lineages in central and western Africa to understand more about deep African lineages seems warranted, and this current study illustrates the broad insights that can sometimes be revealed by very rare lineages.

In conclusion, sequencing of the D0 Y chromosomes and placement of them on a calibrated Y-chromosomal phylogeny identify the most likely model of Y-chromosomal exit from Africa: an origin of the DE lineage inside Africa and expansion out of the C, D, and FT lineages. It suggests an exit time interval that overlaps with the time of Neanderthal admixture estimated from autosomal analyses, and slightly refines it. These findings are consistent with a shared history of Y chromosomes and autosomes, and illustrate how study of Y lineages may lead to general new insights.
